# Relationship between permeability and diffusivity in polyethylene glycol hydrogels

**DOI:** 10.1063/1.5036999

**Published:** 2018-10-04

**Authors:** G. S. Offeddu, E. Axpe, B. A. C. Harley, M. L. Oyen

**Affiliations:** 1 The Nanoscience Centre, Department of Engineering, University of Cambridge, Cambridge CB3 0FF, United Kingdom; 2 Department of Chemical and Biomolecular Engineering, University of Illinois at Urbana−Champaign, 110 Roger Adams Lab., 600 S. Mathews Avenue, Urbana, Illinois 61801, United States

## Abstract

The transport properties of hydrogels largely affect their performance in biomedical applications ranging from cell culture scaffolds to drug delivery systems. Solutes can move through the polymer mesh as a result of concentration gradients in the interstitial fluid or pressure gradients that move the fluid and solutes simultaneously. The relationship between the two modalities of transport in hydrogels can provide insight for the design of materials that can function effectively in the dynamic conditions experienced *in vitro* and *in vivo*, yet this correlation has not been previously elucidated. Here, fluorescence recovery after photobleaching (FRAP) is used to measure the diffusivity of dextran molecules of different size within polyethylene glycol hydrogels. Spherical indentation analyzed in a poroelastic framework is used to measure the permeability to fluid flow of the same hydrogels. It is found that while the diffusivity varies with exp(*ξ*^-2^), where *ξ* is the mesh size of the hydrogels, it also varies with exp(*k*^-1^), where *k* is the intrinsic permeability. For the same hydrogel structure, diffusive transport is affected by the solute size, while convective transport is unaffected. As spherical indentation is a reliable, quick and non-destructive testing method for hydrated soft materials, the relationship provides the means to faster assessment of the transport properties of hydrogels and, ultimately, of their effective use in biomedical applications.

Control of solute transport within hydrogels, polymer networks incorporating large quantities of water, determines the successful use of these materials in biomedical applications. Cells encapsulated in hydrogels must rely on significant transport across the polymer mesh for continuous access to nutrients.^1^ In contrast, when hydrogels are used as drug delivery agents, restricting the mobility of macromolecules may be favorable to allow modulation of the release profile.^2^ In yet other applications, it is the transport specificity of hydrogel membranes that determines their capability for sorting and sensing biomacromolecules.^3^ An understanding of the parameters affecting transport is, therefore, key to the design of hydrogels for specific purposes.

In each application mentioned, the hydrogel may be subjected to forces that produce movement of its interstitial fluid. This is the case in the dynamic environment of the body during drug release or cell culture *in vivo*, as well as during fluid filtration using hydrogel membranes. Convection of the interstitial fluid may increase the transport of cell nutrients or drugs: Indeed, solutes can travel inside hydrogels through diffusion *within* the fluid, by action of a concentration gradient, as well as by convection *with* the fluid, driven by a pressure gradient. Quantification of convective transport in addition to diffusive transport of molecules is, therefore, critical to assess the efficacy of hydrogels as tools for biomedical engineering.

The mobility of solutes by diffusion within the pores is quantified by the diffusion coefficient, *D*_S_, which is some fraction of the diffusion coefficient of the solute in pure liquid, *D*_0_.^4^ The mobility of fluids by convection, in turn, is expressed by the hydraulic permeability, *K*, defined as the ratio between the intrinsic permeability of the material, *k*, and the dynamic viscosity of the fluid, *η*:^5^

$$\begin{array}{c} K=\frac{k}{\eta } \end{array}$$ (1)

The relationship between these two parameters, intrinsic permeability and solute diffusivity, has been previously probed in agarose hydrogels.^6^ Yet, these biopolymer-based materials present intrinsic complexity in their morphology that makes a connection between transport efficiency and structural parameters challenging. Both diffusion and convection in porous media have been shown to depend on the morphological parameters of porous materials in a similar way: Diffusive and convective mobilities increase with the width of the transport paths, *i.e.* the pore size,^4,5^ and decrease with the tortuosity (length) of these paths.^7^ However, the relationship between the two modalities of transport has yet to be elucidated and may allow to predict one from the other, while also producing a more comprehensive characterization of the transport capabilities of hydrogels.

Here, we investigated diffusive and convective transports within polyethylene glycol (PEG) hydrogels. These synthetic materials have been studied extensively for biomedical applications due to their low immunogenicity and protein-binding.^8^ To fabricate the hydrogels, PEG dimethacrylate with a molecular weight, *M*_w_, of 1000 g mol^-1^ (Polysciences Inc., USA) was dissolved in phosphate-buffered saline (PBS) in concentrations of 5, 7, 10 and 25 %w/w. Ammonium persulfate (APS, Sigma Aldrich, UK) was dissolved in distilled water at a concentration of 10 %w/w. Volumes of 150 μL of APS and 75 μL of N,N,N′,N′-tetramethylethylene-diamine (TEMED, Sigma Aldrich, UK) were added simultaneously to the PEG solution, which was then poured into cylindrical molds with diameter of 35 mm. After crosslinking overnight at room temperature, the resulting hydrogels were allowed to swell to equilibrium in PBS over 24 hours, and the increase in volume was measured to yield the volumetric swelling ratio, *Q*_V_.^9^

The hydraulic permeability of PEG hydrogels was measured using a spherical indentation approach we have previously applied to a range of hydrogels and other biomaterials, including polyacrylamide,^10^ gelatin and agar,^11^ microporous freeze-dried collagen scaffolds,^9^ and even biological tissues such as bone and cartilage.^12^ Indentation was conducted with an 8 mm-radius glass spherical tip, using an Instron 5544 universal testing machine (Instron, USA). A ramp-hold profile in displacement was applied, with a ramp of 10 s down to an indentation depth of 0.1 mm, and a hold of 120 s, over which load relaxation took place. A poroelastic framework of analysis was applied as previously described^13^ to obtain *K*, as well as the shear modulus *G*. Testing was performed on two samples per condition, for a total of ten indents per condition. Measurement of the hydraulic permeability *K* also yielded the intrinsic permeability *k*, through knowledge of the viscosity of PBS (8.9 x 10^-4^ Pa s).

The permeability values measured for the hydrogels were linked to the structural features of the materials. The morphology of chemically-crosslinked polymer networks like PEG hydrogels is relatively simple, as the tortuosity is virtually equal to one through the open network. The structure can then be described by one parameter only, the spacing between polymer molecules, or mesh size, *ξ*, which is related to the solid (polymer) volume fraction within the hydrogels, or one minus the porosity available for transport.^14^ This solid fraction, *φ*, can be measured by assessing the dry mass of the hydrogels and by knowledge of the density of the polymer and the fluid. The mesh size is then calculated as:^15^

$$\begin{array}{c} \xi =\varphi^{-\frac{1}{3}}C_{\text{n}}^{\frac{1}{2}}l{\left(\frac{2M_{\text{c}}}{M_{\text{r}}}\right)}^{\frac{1}{2}} \end{array}$$ (2)

where *C*_n_ is a characteristic constant for the polymer characterizing its intrinsic stiffness, equal to four for PEG,^16^ *l* is the length of the atomic bonds along the polymer chain (taken as the average between the oxygen-carbon bond length, 0.143 nm, and the carbon-carbon bond length, 0.154 nm), and *M*_r_ is the molecular weight of the repeating unit, 62.07 g mol^-1^. The parameter *M*_c_ is the molecular weight between crosslinks, a function of *G* and *Q*_V_.^17^ All parameters measured for the hydrogels as part of this study can be accessed through the link to the data repository provided in the Acknowledgments.

Figure 1 shows the relationship between permeability and the two structural parameters. It has been previously shown that the square root of the intrinsic permeability approximates the size of the fluid path, in this case corresponding with *ξ*.^18^ Here, a quadratic relationship was seen to describe well the increase of permeability with mesh size (R^2^ = 0.92, Figure 1.a), except for the hydrogels with largest *ξ*, where the permeability was larger than expected. Similarly, a power relationship with exponent -1.5 is expected for the decrease in permeability with polymer volume fraction,^19^ which was again seen to apply to the materials except for the hydrogel with the largest permeability (R^2^ = 0.96, Figure 1.b). It is possible that for these hydrogels (5 %w/w), the permeability was overestimated due to larger time-dependent deformations than cannot be accounted for by poroelastic mechanisms alone. Indeed, for these hydrogels the load relaxed by 68 ± 16 %, compared to 20 ± 3 % on average for the other more-crosslinked materials, implying that additional viscoelastic phenomena possibly take place at small PEG concentrations. The calculated exponents when all four hydrogel conditions were taken into account were 1.55 ± 0.38 (R^2^ = 0.59) for the mesh size, and -2.07 ± 0.46 (R^2^ = 0.62) for the polymer volume fraction (all fitting and statistical analysis throughout the study was performed using the software OriginPro 2016).

**FIG. 1.**
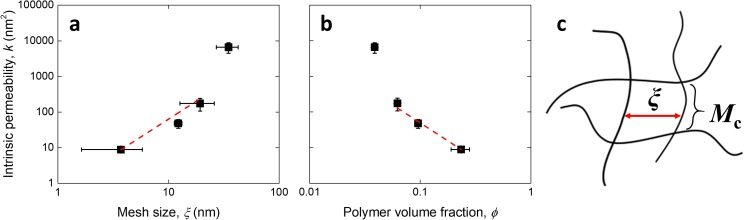
Relationship between intrinsic permeability of PEG hydrogels and their (a) mesh size and (b) polymer volume fraction. The theoretical power curves of exponents 2 and -1.5 are shown for comparison in (a) and (b), respectively. All values are reported as mean ± standard deviation. A schematic diagram for the mesh size and molecular weight between crosslinks is portrayed in (c).

The diffusivity of fluorescein isothiocyanate (FITC)-labelled dextrans of molecular weights 4, 20 and 2000 kDa (Sigma Aldrich, UK), corresponding to hydrodynamic radii of 1.9, 3.5 and 19.4 nm (calculated through the Stokes-Einstein approximation^20^), was assessed by fluorescence recovery after photobleaching (FRAP). Briefly, FITC-dextrans were dissolved in the PEG aqueous solution at a concentration of 0.5 mg mL^-1^, after which the hydrogels were prepared as described above. FRAP was conducted at room temperature on a Leica TCS SP8 Confocal microscope (Germany) using a 488 nm laser to bleach a circular spot for ten frames (189 ms per frame), to then record the photo-recovery over 50 to 300 frames. The diffusion coefficient was measured by using the following equation:^21^

$$\begin{array}{c} D_{\text{S}}=\frac{w^{2}}{\tau } \end{array}$$ (3)

where *w* is the width of the circular spot (15 μm) and *τ* the recovery time, yielded by the microscope software, Leica Application Suite X. Ten FRAP experiments per condition were performed.

The resulting diffusivities, expressed as the ratio *D*_S_ ⁄ *D*_0_, where *D*_0_ is the diffusion coefficient of the particular solute in PBS alone as taken from Ref. 20, are reported in Figure 2. Various models exist to describe the relationship between the structural parameters of hydrogels and diffusivity, reviewed in Ref. 22. The polymer mesh can be thought of as a sieve for the solutes that effectively obstructs the passage of large molecules, increasing the mean path length. The obstruction model proposed by Amsden^23^ considers the relationship between mesh size *ξ*, solute hydrodynamic radius *r*_h_, and polymer fiber radius *r*_f_ so that:

$$\begin{array}{c} \frac{D_{\text{S}}}{D_{0}}=\exp\left(-\frac{\pi {\left(r_{\text{h}}\;+\;r_{\text{f}}\right)}^{2}}{{\left(\xi \;+\;2r_{\text{f}}\right)}^{2}}\right) \end{array}$$ (4)

Figure 2.a shows that the proportionality between diffusivity and exp(-*ξ*^-2^) describes reasonably well the trend in values measured within PEG hydrogels other than for 20 kDa dextran, as possibly due to a broader size distribution compared to the other solutes that results in the larger experimental variability observed (*R*^2^ = 0.98, 0.66, 0.85 for 4, 20, 2000 kDa). Alternatively, diffusion through the hydrogel can be considered as a process whereby in order to translate, solutes occupy free volume between solvent molecules. Lustig and Peppas developed a diffusivity model that takes into account the decrease in available free volume when the polymer mesh is present.^24^ According to the model, the diffusivity varies with exp[*φ* ⁄ (1 − *φ*)], a proportionality seen to better describe the measured diffusion coefficient values in PEG hydrogels (Figure 2.b, *R*^2^ = 0.99, 0.87, 0.99 for 4, 20, 2000 kDa).

**FIG. 2.**
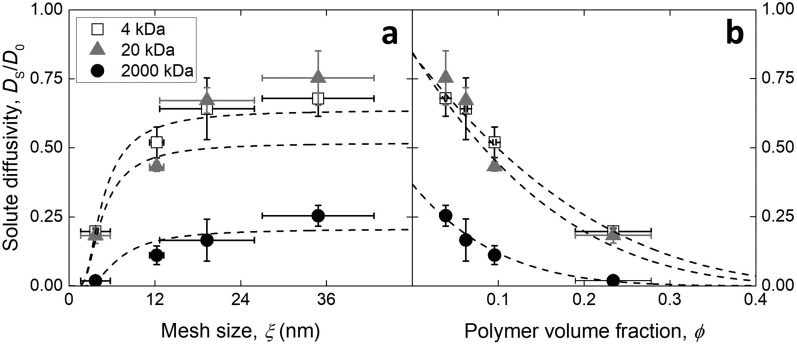
Diffusivity of dextrans of three molecular sizes within PEG hydrogels characterized by a varying (a) mesh size or (b) polymer volume fraction. The dashed lines represent the curve fits performed. Values reported as mean ± standard deviation.

In order to probe the relationship between convection and diffusion of different dextrans within a particular material structure, hydrogels (7% w/w) containing the three molecular weight solutes were indented to measure the hydraulic permeability. An obstruction factor, in the form of the ratio between mesh size for the hydrogels and solute hydrodynamic diameter, *ξ*/2*r*_h_, was calculated and is reported in Figure 3.a. As schematized in Figure 3.b, for these hydrogels the solute sizes varied from smaller (4, 20 kDa) to larger (2000 kDa) compared to the mesh size of 7 %w/w hydrogels. Despite the variation in obstruction factor for the different dextrans, the hydraulic permeability was found to be constant (the gradient of the fitted line is not significantly different from zero, *p*-value = 0.16) and consistent with that of the samples free of solutes (Figure 3.c). As the obstruction of pores by the dextrans would result in fewer paths available for fluid flow and, consequently, a smaller hydraulic permeability, this result suggests that the convective transport of dextrans is unaltered irrespective of their size relative to the mesh. The solutes may be pushed through the polymer mesh by the action of the hydrostatic pressure, as was observed in the case of cartilage tissue for the transport of solutes larger than the spacing between extracellular matrix components.^25^

**FIG. 3.**
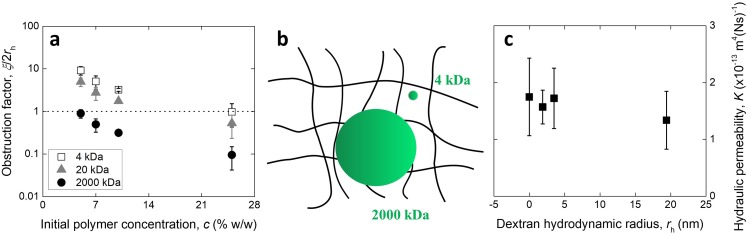
(a) Variation in the ratio between mesh size and solute hydrodynamic diameter for three dextran molecules in three PEG hydrogels of varying precursor concentration, schematically depicted in (b). (c) Hydraulic permeability measured for 7 %w/w PEG hydrogels containing dextrans of different sizes. Values reported as mean ± standard deviation.

As noted above, the square root of the intrinsic permeability can be directly taken as the size of the transport paths, without introduction of further constants. It follows that substitution of √*k* for *ξ* in the model proposed by Amsden should also fit the results. In Figure 4.a it is shown that this was the case, and the proportionality fit was overall of better quality compared to that using the mesh size (*R*^2^ = 0.99, 0.82, 0.95 for 4, 20, 2000 kDa). The diffusivity for the various dextran sizes in PEG hydrogels was, therefore, calculated using:

$$\begin{array}{c} \frac{D_{\text{S}}}{D_{0}}=\exp\left(-\frac{\pi {\left(r_{\text{h}}\;+\;r_{\text{f}}\right)}^{2}}{{\left(\sqrt{k}\;+\;2r_{\text{f}}\right)}^{2}}\right) \end{array}$$ (5)

which resulted in better prediction of the results compared to Eq. 4 (Figure 4.b, the line gradients in the parity plot were 1.5 ± 0.06 and 1.39 ± 0.05 for the *ξ* and √*k* predictions, respectively, taking *r*_f_ = 0.51 nm^26^). The points calculated for the 5 %w/w hydrogels are highlighted, for which the permeability was possibly overestimated and the prediction using the mesh size was better in all cases. The overestimation of the diffusion coefficient of dextrans by both methods may be due to unaccounted interaction phenomena between the molecules and the polymer matrix. Indeed, dextrans do not possess the spherical geometry implied by their hydrodynamic radius, but are instead long molecules able to unravel as they translate through the hydrogel mesh. The theoretical model for diffusion in hydrogels developed by Amsden, on which the permeability relationship proposed here is based, has been validated for a number of hydrogels other than PEG, including polyacrylamide, polyvinyl alcohol and polyethylene oxide.^23^ At the same time, the correlation between mesh size and the square of the permeability is based on a physical model^18^ that found validation in our results. Thus, it is expected that the relationship proposed herein between permeability and diffusivity in PEG hydrogels can be applied to other hydrogel systems. Nevertheless, further studies involving these and other types of hydrogels and solutes will be necessary to ascertain the relationship between solute properties and convective flux in hydrogels.

**FIG. 4.**
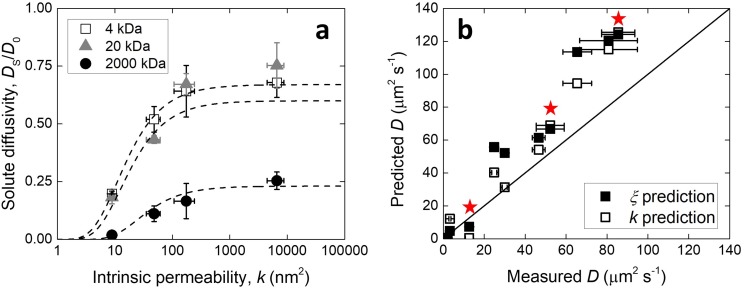
Diffusivity of dextran molecules within PEG hydrogels as a function of (a) their intrinsic permeability, or (b) compared to the predicted values using the mesh size or the square of the intrinsic permeability as the size of the transport paths. In (a) the dashed lines represent the fit described in-text. In (b) the star symbols indicate the samples for which the intrinsic permeability was overestimated. Values reported as mean ± standard deviation.

In summary, we applied an indentation-based methodology to measure directly the intrinsic permeability of PEG hydrogels, a parameter that is straightforwardly related to the mesh size of these materials. As the size of the transport paths is thought to affect the diffusive transport of solutes within hydrogels, we attempted to predict the diffusivity of dextrans within these materials through the use of the intrinsic permeability values measured. The prediction was significantly more accurate than that performed using mesh size values, which calculation requires the knowledge and measurement of a number of other parameters. The intrinsic permeability of PEG and other hydrogels can be measured directly using the method described here, as well as others.^27^ In particular, as spherical indentation is a fast, non-destructive method that can be performed at multiple scales,^17,28^ it may allow for quick assessment of both diffusive and convective modes of transport within hydrogels.

## References

[1] J. L. Drury and D. J. Mooney, Biomaterials 24, 4337 (2003). 10.1016/s0142-9612(03)00340-5 12922147

[2] A. Vashist, A. Vashist, Y. K. Gupta, and S. Ahmad, J. Mater. Chem. B 2, 147 (2014). 10.1039/c3tb21016b 32261602

[3] S. Ladet, L. David, and A. Domard, Nature 452, 76 (2008). 10.1038/nature06619 18322531

[4] N. A. Hadjiev and B. G. Amsden, J. Contr. Rel. 199, 10 (2015). 10.1016/j.jconrel.2014.12.010 25499554

[5] F. J. O’Brien, B. A. Harley, I. V. Yannas, L. J. Gibson, and P. J. Prendergast, Technol. Health Care 15, 3 (2007). 17264409

[6] K. B. Kosto and W. M. Deen, AIChE J. 50, 2648 (2004). 10.1002/aic.10216

[7] N. Epstein, Chem. Eng. Sci. 44, 777 (1989). 10.1016/0009-2509(89)85053-5

[8] C. C. Lin and K. S. Anseth, Pharma. Res. 26 (2009). 10.1007/s11095-008-9801-2 PMC5892412 19089601

[9] G. S. Offeddu, J. C. Ashworth, R. E. Cameron, and M. L. Oyen, Acta Biomater. 41, 193 (2016). 10.1016/j.actbio.2016.05.024 27255358

[10] M. Galli, K. S. C. Comley, T. A. V. Shean, and M. L. Oyen, J. Mater. Res. 24, 973 (2009). 10.1557/jmr.2009.0129

[11] D. G. T. Strange and M. L. Oyen, J. Mech. Behav. Biomed. Mater. 11, 16 (2012). 10.1016/j.jmbbm.2011.10.003 22658151

[12] M. L. Oyen, T. A. V. Shean, D. G. T. Strange, and M. Galli, J. Mater. Res. 27, 245 (2012). 10.1557/jmr.2011.322

[13] M. L. Oyen, J. Mater. Res. 23, 1307 (2008). 10.1557/jmr.2008.0156

[14] K. Nakamura, R. J. Murray, J. I. Joseph, N. A. Peppas, M. Morishita, and A. M. Lowman, J. Control. Rel. 95, 589 (2004). 10.1016/j.jconrel.2003.12.022 15023469

[15] P. X. Ma and J. H. Elisseeff, Scaffolding in tissue engineering (Taylor & Francis, 2005).

[16] S. P. Zustiak and J. B. Leach, Biomacromolecules 11, 1348 (2010). 10.1021/bm100137q 20355705 PMC3050024

[17] G. S. Offeddu, I. Mela, P. Jeggle, R. M. Henderson, S. K. Smoukov, and M. L. Oyen, Sci. Rep. 7, 42948 (2017). 10.1038/srep42948 28230077 PMC5322396

[18] G. W. Scherer, J. Sol-Gel Sci. Tech 1, 285 (1994). 10.1007/bf00486171

[19] P. J. deGennes, Scaling concepts in polymer physics (Cornell University Press, 1979).

[20] P. Gribbon and T. E. Hardingham, Biophys. J. 75, 1032 (1998). 10.1016/s0006-3495(98)77592-7 9675204 PMC1299777

[21] F. Brandl, F. Kastner, R. M. Gschwind, T. Blunk, J. Tessmar, and A. Gopferich, J. Control. Release 142, 221 (2010). 10.1016/j.jconrel.2009.10.030 19887092

[22] B. Amsden, Macromolecules 31, 8382 (1998). 10.1021/ma980765f

[23] B. Amsden, Macromolecules 32, 874 (1999). 10.1021/ma980922a

[24] S. R. Lustig and N. A. Peppas, J. Appl. Polym. Sci. 36, 735 (1988). 10.1002/app.1988.070360401

[25] B. P. O’Hara, J. P. G. Urban, and A. Maroudas, Annals Rheum. Dis. 49, 536 (1990). 10.1136/ard.49.7.536 PMC1004145 2383080

[26] V. Hagel, T. Haraszti, and H. Boehm, Biointerphases 8, 1 (2013). 10.1186/1559-4106-8-36 24706145

[27] F. Pennella, G. Cerino, D. Massai, D. Gallo, G. Falvo D’Urso Labate, A. Schiavi, M. A. Deriu, A. Audenino, and U. Morbiducci, Annals Biomed. Eng. 41, 2017 (2013). 10.1007/s10439-013-0815-5 23612914

[28] G. S. Offeddu, J. C. Ashworth, R. E. Cameron, and M. L. Oyen, J. Mech. Behav. Biomed. Mater. 42, 19 (2015). 10.1016/j.jmbbm.2014.10.015 25460922

